# Supplementary material for: Classification of Subgroups with Immune Characteristics Based on DNA Methylation in Luminal Breast Cancer
Source: Int J Mol Sci. 2022 Oct 22;23(21):12747. doi: 10.3390/ijms232112747 (PMC9658742; doi:10.3390/ijms232112747)
Supplement: Supplementary file 1 [file ijms-23-12747-s001.zip › Supplementary.pdf]

Supplementary Information

Classification of subgroups with immune characteristics based on DNA methylation in luminal breast cancer

Index

Supplementary Methods 2

The selection of KMscore... 2

“Boruta” feature selection.. 2

Fig. S1 BC heterogeneity based on DNA methylation. 3

Fig. S2 Differential genes and gene function enrichment pathways in luminal breast cancer and normal. 4

Fig. S3 SVM classifier performance of luminal A/B.. 5

Fig.S4 The selection of best number of clusters. 5

Fig. S5 Single cell type and proportion of cell infiltration in subtypes......................... 6

Fig. S6 Survival curve of immunophenotypes from TCGA in luminal breast cancer. . 6

Fig. S7 Relationship of subtypes in luminal breast cancer and immune cell lysis activity (CYT). 7

Fig. S8 Intersection between immune genes and differential DNA methylation genes. 7

Fig. S9 Concordance of key genes and specific immune genes of luminal cases in TCGA and GEO datasets.. 8

Table S4 TILs pattern enriched in luminal A/B subgroups 8

Supplementary Methods

The selection of KMscore

In order to select the optimal KMscore for the screening of key modules, we compared the quantiles of different KMscores, which were 60%, 65%, 70%, 75%, 80%, and 85%. By selecting different quantiles number to get different key genes. Based on hierarchical clustering, the luminal A/B samples were divided into different subgroups. Subgroups distinguished by “key genes” were considered meaningful when survival were significant different between subgroups. As the threshold decreased, that was, the number of key genes increased, the *P* value of the difference in survival between subgroups increased (Fig. S10)

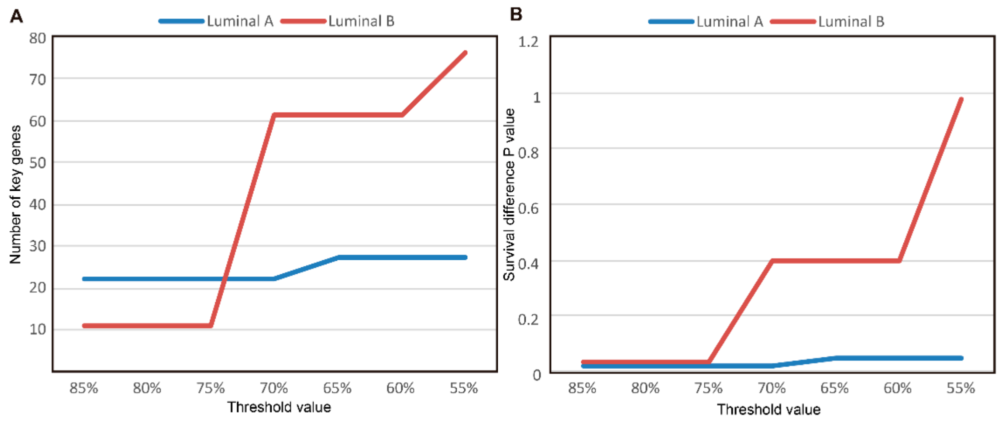

Figure S10 Comparison of KMscore threshold selection for key modules and subgroup survival differences**. (A)** KMscore threshold and the number of “key genes” screened. **(B)** KMscore threshold and the *P* value in subgroup survival differences.

“Boruta” feature selection

The consistency test was used to verify that feature selection was not affected by samples. We performed five-fold cross-validation on the samples of luminal A/B respectively, and then applied "Boruta" feature selection in each fold. We checked the consistency of the results in the five-fold feature selection. The *P* value was applied for the result of consistency test. The *P* value of the consistency test was less than 0.05, which showed that there was no difference in the feature gene sets of the five-fold. (P<0.05) (Fig. S11).

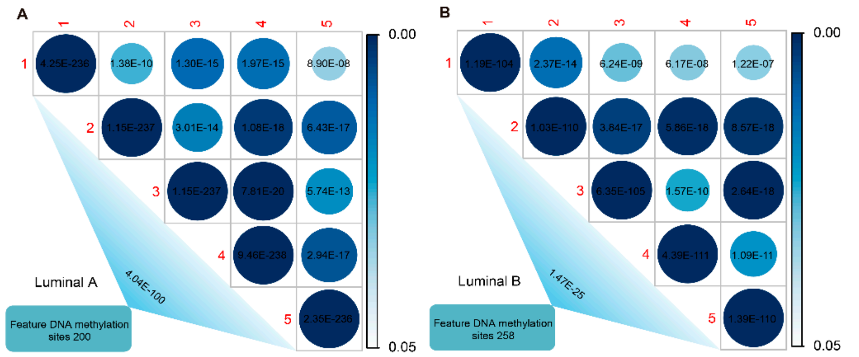

Figure S11 Consistency test between five-fold and training samples based on “Boruta” algorithm. **(A)** Consistency test between five-fold and training samples based on “Boruta” algorithm in luminal A. **(B)** Consistency test between five-fold and training samples based on “Boruta” algorithm in luminal B.

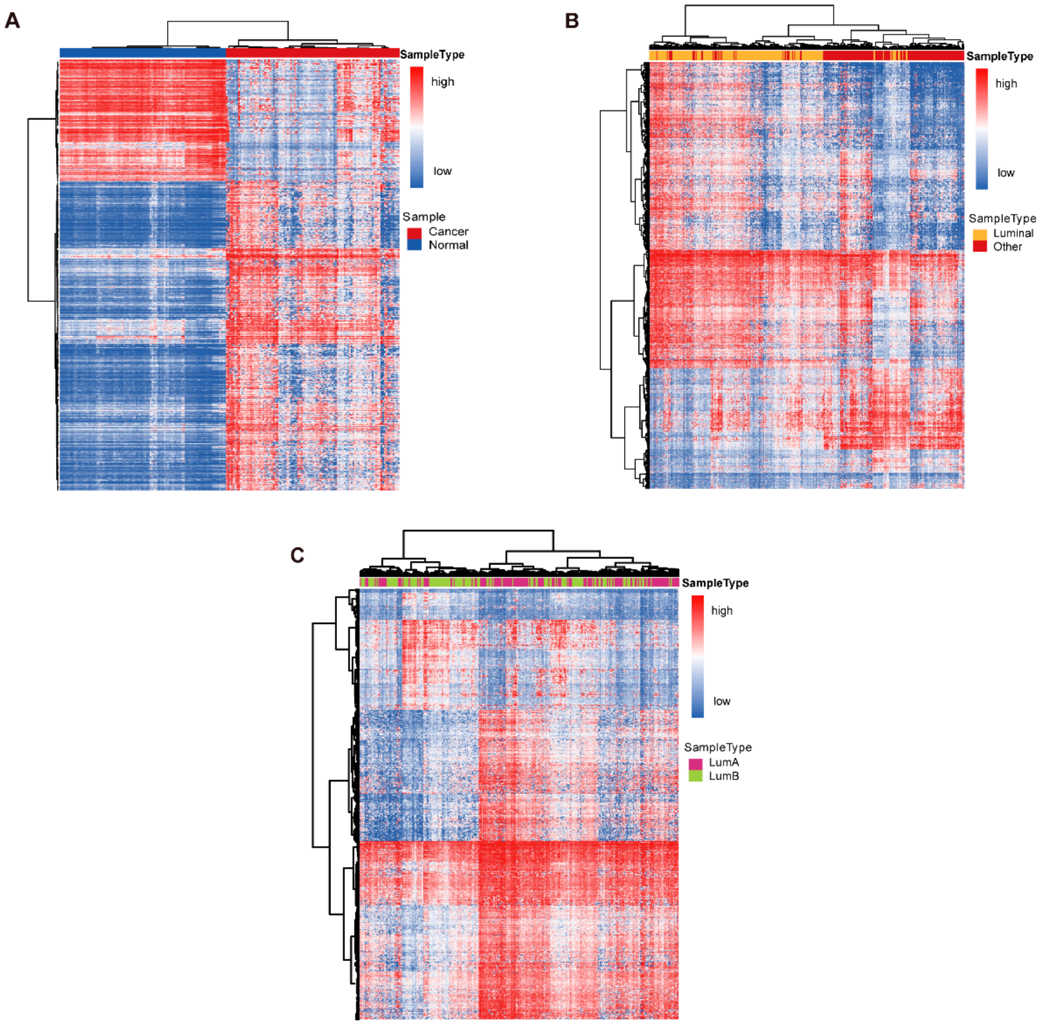

Figure S1 BC heterogeneity based on DNA methylation. **(A)**. Heatmap of differential DNA methylation genes for cancer and normal samples. **(B)** Differential DNA methylation in luminal and other subgroups **(C)** Differential DNA methylation in luminal A and luminal B.

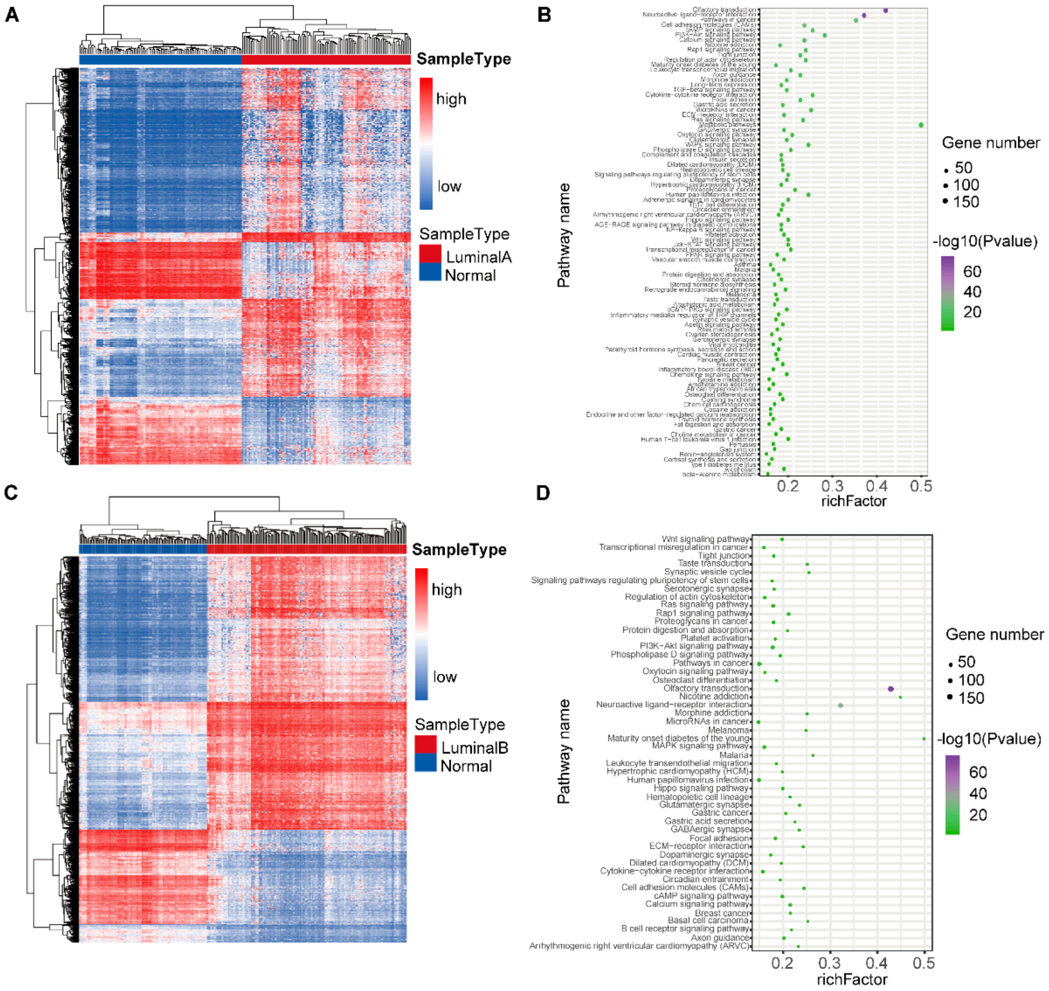

Figure S2 Differential genes and gene function enrichment pathways in luminal breast cancer and normal. **(A)** Luminal A breast cancer and normal differential genes. **(B)** Luminal A breast cancer and normal differential gene function enrichment pathways. **(C)** Luminal B breast cancer and normal differential genes. **(D)** Luminal B breast cancer and normal differential gene function enrichment pathways.

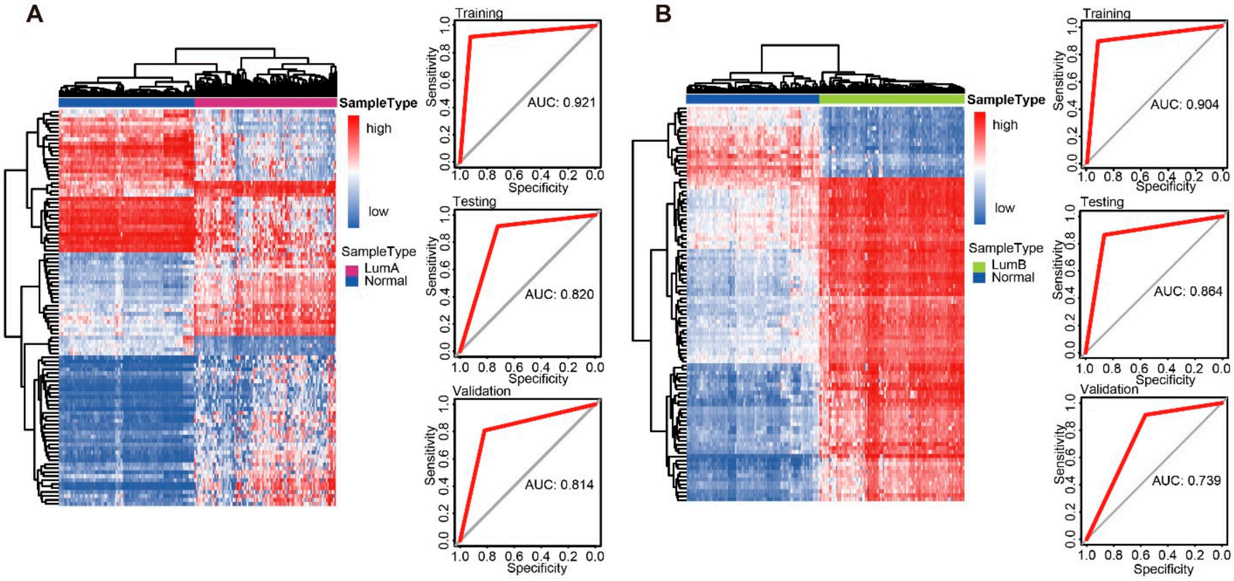

Figure S3 SVM classifier performance of luminal A/B **(A)** Heatmap of differentially DNA methylation sites and svm classifier performance on training, validation and test sets in luminal A**. (B)** Heatmap of differentially DNA methylation sites and SVM classifier performance on training, validation and test sets in luminal B**.**

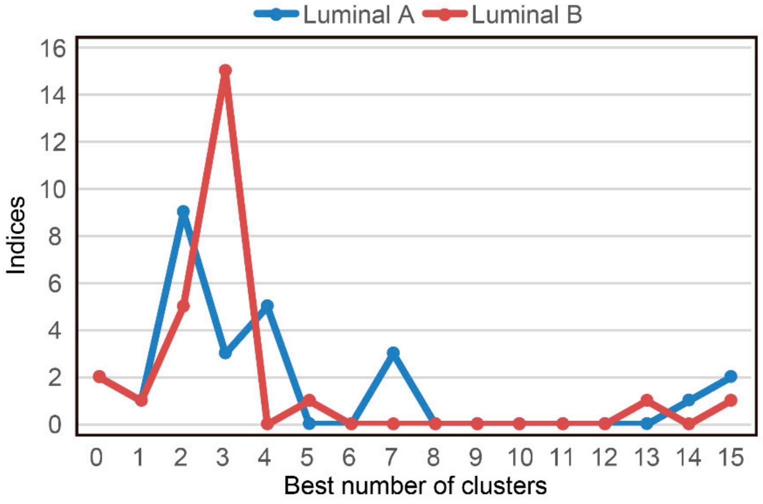

FigureS4 The selection of best number of clusters.

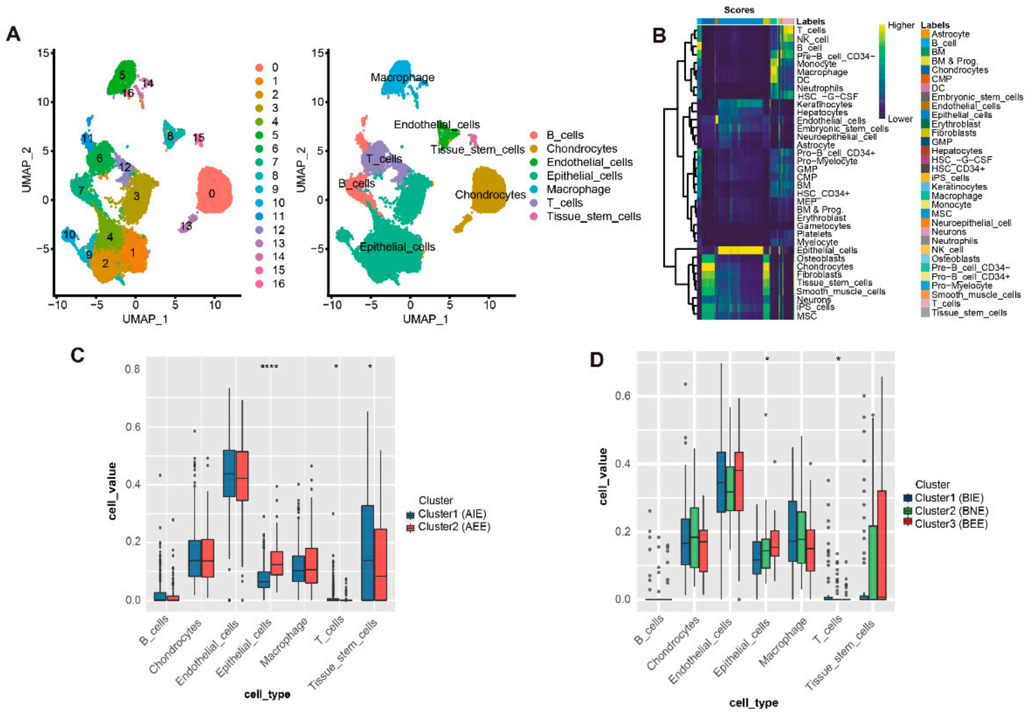

Figure S5 Single cell type and proportion of cell infiltration in subtypes. **(A)** Seurat single-cell clustering labels. **(B)** Single-cell annotation heatmap. **(C)** Cell proportions of luminal A. **(D)** Cell proportions of luminal B. (*, P < 0.05. **, P < 0.01. ***, P < 0.001. ****, P<0.0001.)

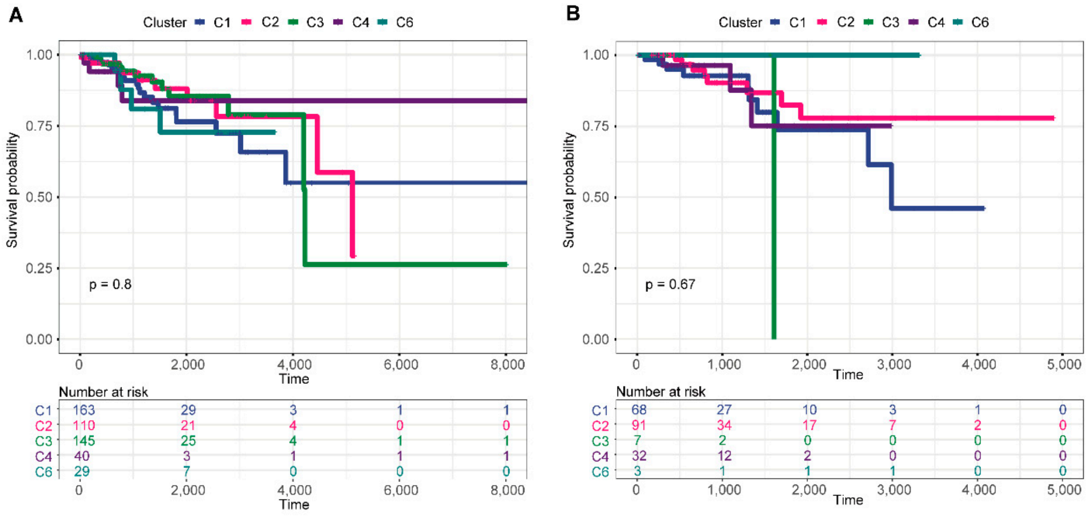

Figure S6 Survival curve of immunophenotypes from TCGA in luminal breast cancer. **(A)** Survival curve of immunophenotypes in luminal A. **(B)** Survival curve of immunophenotypes in luminal B.

C1, C2, C3, C4, C6 were the different immune subtypes from TCGA.

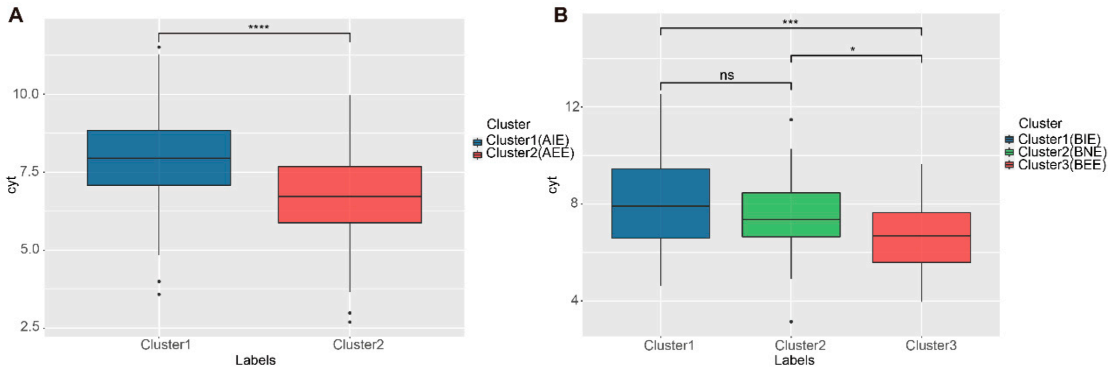

Figure S7 Relationship of subtypes in luminal breast cancer and immune cell lysis activity (CYT). **(A)** Differences of luminal A subtypes in CYT. **(B)** Differences of luminal B subtypes in CYT.

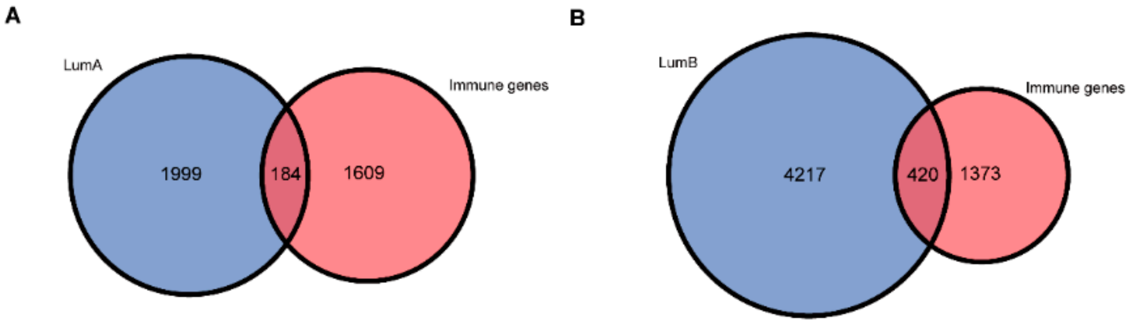

Figure S8 Intersection between immune genes and differential DNA methylation genes. **(A)** Intersection genes between immune genes and differential DNA methylation genes in luminal A. **(B)** Intersection genes between immune genes and differential DNA methylation genes in luminal B.

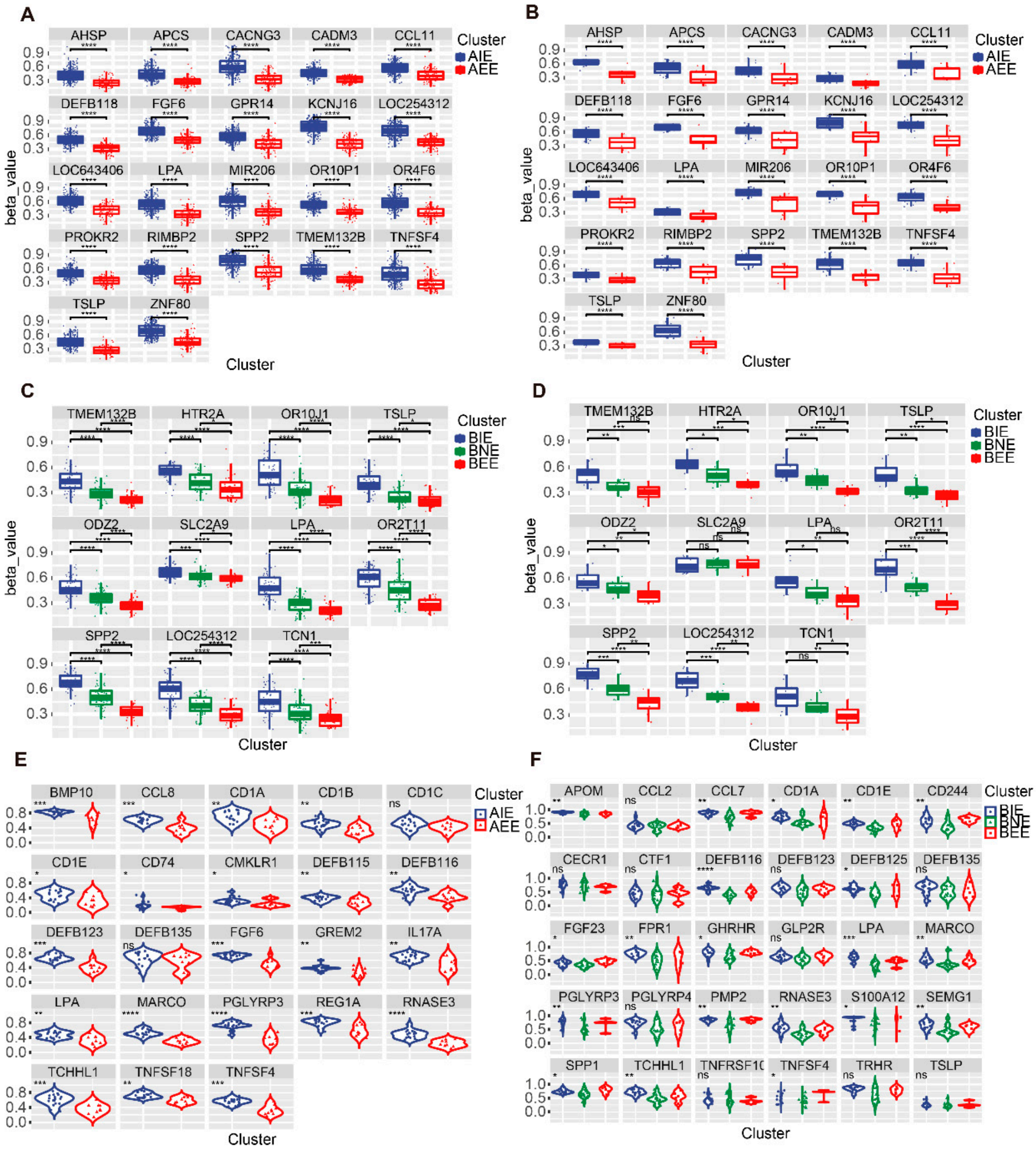

Figure S9 Concordance of key genes and specific immune genes of luminal cases in TCGA and GEO datasets. **(A)** DNA methylation levels of key genes of luminal A subtypes in TCGA. **(B)** DNA methylation levels of key genes of luminal A subtypes in GEO. **(C)** DNA methylation levels of key genes of luminal B subtypes in TCGA. **(D)** DNA methylation levels of key genes of luminal B subtypes in GEO. **(E)** DNA methylation levels of immune genes in luminal A on GEO. **(F)** DNA methylation levels of immune genes in luminal B on GEO. (*, $P < 0.05$. **, $P < 0.01$. ***, $P < 0.001$. ****, $P<0.0001$.)

Table S4 TILs pattern enriched in luminal A/B subgroups

Global_Pattern	Subgroup	Sample	*P* value
Brisk Band-like	AIE	21	
	AEE	8	0.965
	BIE	11	
	BNE	17	

	BEE	2	**0.009**
Brisk Diffuse	AIE	48	
	AEE	16	1
	BIE	17	
	BNE	10	
	BEE	6	**0.05**
Non-Brisk Focal	AIE	91	
	AEE	38	0.212
	BIE	14	
	BNE	14	
	BEE	20	**0.017**
Non-Brisk Multifocal	AIE	50	
	AEE	8	**0.033**
	BIE	4	
	BNE	10	
	BEE	8	0.198
None	AIE	3	
	AEE	3	
